# The Specificity of the Link Between Alexithymia, Interoception, and Imitation

**DOI:** 10.1037/xhp0000310

**Published:** 2016-11

**Authors:** Sophie Sowden, Rebecca Brewer, Caroline Catmur, Geoffrey Bird

**Affiliations:** 1 MRC Social, Genetic and Developmental Psychiatry Centre, Institute of Psychiatry, Psychology and Neuroscience, King’s College London; 2 School of Psychology, University of East London; 3 Department of Psychology, Institute of Psychiatry, Psychology and Neuroscience, King’s College London; 4 MRC Social, Genetic and Developmental Psychiatry Centre, Institute of Psychiatry, Psychology and Neuroscience, King’s College London, and Institute of Cognitive Neuroscience, University College London

**Keywords:** alexithymia, interoception, imitation-inhibition, self–other processing

## Abstract

Alexithymia is a subclinical condition traditionally characterized by difficulties identifying and describing one’s own emotions. Recent formulations of alexithymia, however, suggest that the condition may result from a generalized impairment in the perception of all bodily signals (“interoception”). Interoceptive accuracy has been associated with a variety of deficits in social cognition, but recently with an improved ability to inhibit the automatic tendency to imitate the actions of others. The current study tested the consequences for social cognition of the hypothesized association between alexithymia and impaired interoception by examining the relationship between alexithymia and the ability to inhibit imitation. If alexithymia is best characterized as a general interoceptive impairment, then one would predict that alexithymia would have the same relationship with the ability to control imitation as does interoceptive accuracy. Forty-three healthy adults completed measures of alexithymia, imitation-inhibition, and as a control, inhibition of nonimitative spatial compatibility. Results revealed the predicted relationship, such that increasing alexithymia was associated with an improved ability to inhibit imitation, and that this relationship was specific to imitation-inhibition. These results support the characterization of alexithymia as a general interoceptive impairment and shed light on the social ability of alexithymic individuals—with implications for the multitude of psychiatric, neurological, and neurodevelopmental disorders associated with high rates of alexithymia.

The ability to distinguish and control representations of the “self” and of “others” is important in almost all social contexts. For example, in order to avoid compulsive imitation of the actions of others one must accurately distinguish between other-related motor programs and those belonging to the self, and then control those representations such that representation of one’s own motor program is enhanced and the other’s inhibited. Similarly, when attempting to represent another’s false belief, one must distinguish the mental state of the other from one’s own, and then inhibit representation of one’s own mental state and enhance that of the other (de Guzman, Bird, Banissy, & Catmur, 2016; Santiesteban et al., 2012; Sowden & Shah, 2014).

The cognitive processes supporting the control of self- and other-related representations are largely unknown, although it is possible that common processes are recruited across motor, cognitive, and affective domains (Spengler, Bird, & Brass, 2010). Causal evidence for such a link was provided by Santiesteban and colleagues (2012), who demonstrated that training to inhibit imitation resulted in an improved ability to take the visual perspective of another, that is, participants were better able to separate their own and another’s visual perspective, and selectively represent that of the other. Similar evidence was provided by de Guzman and colleagues, who demonstrated that training to inhibit imitation enhanced empathic responses to another’s pain (de Guzman et al., 2016). A mechanism of self–other control has been suggested to explain results such as these, which enables selective representation of self- or other-relevant representations such that the self is enhanced and the other inhibited (e.g., in the case of inhibiting imitation), or the other enhanced and the self inhibited (in the case of perspective taking or empathy for pain), according to task demands (Hogeveen et al., 2015; Happé, Cook, & Bird, in press).

Recent computational models within the predictive coding framework suggest that the ability to distinguish between self and other is dependent upon interoception, the perception of the internal state of one’s own body (Seth, 2013; Quattrocki & Friston, 2014). These theories suggest that interoceptive information is used to build representations which correspond to one’s “sentient, feeling self” (Seth, 2013). Good awareness of interoceptive cues is therefore thought to be crucial for the awareness of one’s own body, and the representation of oneself as distinct from others. Lower interoceptive accuracy would therefore be expected to result in a reduced ability to distinguish between self and other.

One study is notable, however, in that its results argue against such a relationship between interoceptive accuracy and self–other processing. Ainley, Brass, and Tsakiris (2014) investigated the relationship between interoceptive accuracy and the ability to inhibit imitation. As described previously, when inhibiting the automatic tendency to imitate, one must distinguish one’s own motor intention from that of the other, and then enhance representation of the self and inhibit representation of the other (Brass, Derrfuss, & von Cramon, 2005; Brass, Ruby, & Spengler, 2009; Spengler, Brass, Kühn, & Schütz-Bosbach, 2010; Spengler et al., 2010). Rather than the predicted positive relationship between interoceptive accuracy and ability to inhibit imitation, Ainley et al. (2014) found a negative relationship such that those with superior interoceptive accuracy had greater difficulty inhibiting the tendency to imitate, suggesting poorer ability to distinguish between self and other.

It has recently been suggested that alexithymia, a subclinical condition characterized by an inability to identify and describe one’s own emotions (Sifneos, 1973), is best characterized as a generalized impairment of interoception (Brewer, Happé, Cook, & Bird, 2015; Bird & Viding, 2014). This claim is supported by recent demonstrations of lower accuracy in detecting one’s heartbeat in those with high levels of alexithymia (Herbert, Herbert, & Pollatos, 2011; Shah, Hall, Catmur, & Bird, 2016), along with reduced accuracy when reporting one’s degree of physiological arousal (Gaigg, Cornell, & Bird, in press), as well as a failure to recognize nonaffective interoceptive states such as fatigue, temperature, hunger, and satiety (Brewer, Cook, & Bird, in press). If the hypothesis that alexithymia is characterized by reduced interoceptive accuracy is correct, one would therefore expect the impact of alexithymia to be consistent with that of interoceptive accuracy. In other words, if alexithymia is the result of generally lower interoceptive accuracy, then one would expect it to be associated with the same pattern of abilities and impairments that have previously been demonstrated to be associated with interoceptive accuracy. This is the focus of the current study.

## The Current Study

The results obtained by Ainley et al. (2014) are of theoretical importance; if greater interoceptive accuracy is associated with reduced ability to inhibit imitation, then current theoretical models linking interoception to improved self–other distinction require revision. For the present purposes, however, they provide an opportunity to investigate the functional consequences of the proposed link between alexithymia and interoception. If high levels of alexithymia reflect reduced interoceptive accuracy, then one would expect increasing alexithymia to predict an increased ability to inhibit imitation, as found by Ainley et al. (2014) for interoception.

In addition, the current study is able to investigate the specificity of the link between alexithymia and the inhibition of imitation. Ainley et al. (2014) used a test of imitation in which participants are required to perform either an index- or middle-finger lift. Simultaneously with the participant’s response, a hand presented on the computer screen performed the same action (“imitatively compatible trials”) or a different action (“imitatively incompatible trials”). The reaction time (RT) difference between compatible and incompatible trials is taken as an index of the participant’s ability to inhibit the automatic tendency to imitate the action of the computer hand. In the version of the task used by Ainley et al. (2014), however, imitatively compatible stimuli (e.g., an observed middle-finger lift when a middle-finger lift is required) were also spatially compatible with the required response. There is now ample evidence (Catmur & Heyes, 2011; Cho & Proctor, 2004; Sowden & Catmur, 2015) that stimuli presented on the same side of space as a required response, regardless of their identity, prompt faster responses than those on the opposite side of space. When using such a design, it is impossible to distinguish spatial from imitative compatibility. We therefore utilized a paradigm able to distinguish between imitative and spatial compatibility (Sowden & Catmur, 2015). If effects of interoception and, by hypothesis, alexithymia, are specific to imitation-inhibition, and not more generally to nonimitative stimulus–response compatibility, then we should find effects of alexithymia on imitative, but not spatial, compatibility.

### Method

#### Ethics

The experiment was approved by the local research ethics committee and was performed in accordance with the principles of the Helsinki Declaration (World Medical Association, 2013). All participants provided written informed consent and were aware they could withdraw at any time.

#### Participants

Forty-three healthy adult participants (20 males, mean age = 29.6 years, standard deviation [*SD*] = 12.4) were recruited via King’s College London research recruitment systems. All reported being right-handed, with normal or corrected-to-normal vision, and were typically staff or students affiliated with King’s College London. One further participant was excluded prior to data analysis as that participant made more than 15% errors on the imitation-inhibition task (Sowden & Catmur, 2015).

#### Imitation-inhibition task

**Figure 1 fig1:**
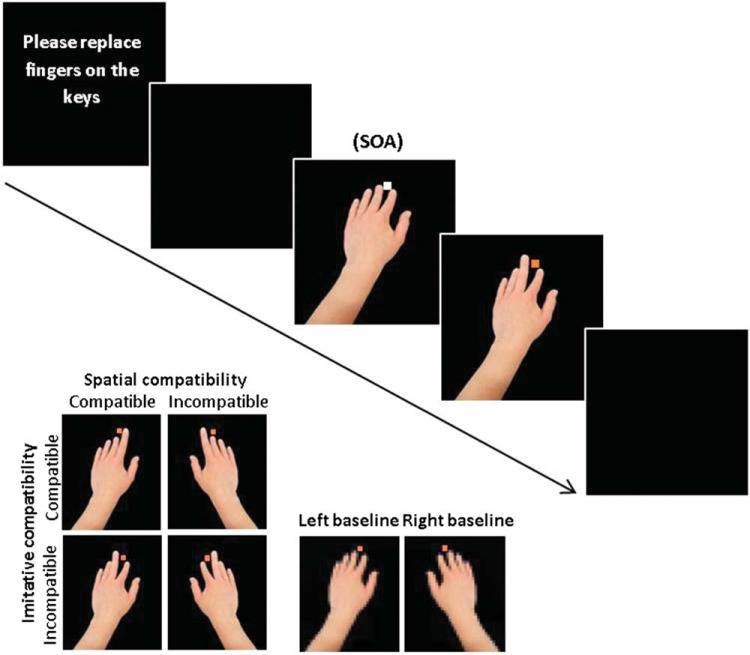
Example of one full trial in the experiment and of the task-irrelevant hand stimuli. Stimulus onset asynchronies (SOA) were 1,600, 2,000, or 2,400 ms. Labels denote spatial and imitative compatibility of stimuli on the standard trials, illustrating the 2 × 2 design, and left- or right-hand stimuli on the baseline trials, when the orange (dark) square indicates a required index-finger lift. When a middle-finger lift is required, levels of spatial and imitative compatibility are each reversed. For this response mapping, the trial illustrated is imitatively incompatible and spatially compatible, whereas for the response mapping for which orange indicates a middle-finger lift, it is imitatively compatible and spatially incompatible. See the online article for the color version of this figure.

The stimuli used in the imitation-inhibition task were identical to those used by Sowden and Catmur (2015), presented in color on a black background (see Figure 1 for full trial and stimuli illustrations), on a 15.6-inch LCD laptop screen via E-Prime2 (Psychology Software Tools, Sharpsburg, PA). Images of human right and left hands served as the task-irrelevant stimuli (horizontal visual angle of 6.5°), consisting of static hands (where all fingers were at rest) and, on standard trials, hands for which the index or middle fingers were in a lifted position (subtending vertical visual angles of 9.4° and 9.2°, respectively). The immediate transition from a static hand to a finger-lift image produces apparent motion of the finger (Press, Bird, Flach, & Heyes, 2005). On baseline trials the static hand was replaced by a pixelated hand which was designed not to elicit spatial or imitative compatibility effects, but precisely matched the timing of the task-irrelevant movement in standard trials.[Fig-anchor fig1]

Task-relevant discriminative stimuli which indicated the participant’s required response were orange or purple squares (occupying 0.2° visual angle). A white square of identical dimensions, positioned between the index and middle fingers of the static hand, functioned as a fixation point. Allocation of discriminative stimuli to response options (index- or middle-finger lift) was counterbalanced across participants, with purple and orange squares indicating whether the participant should lift their index finger (from the *N* key) or middle finger (from the *M* key) on each trial.

Participants sat approximately 80 cm from the screen, placing their right arm (in the same orientation as the hand stimuli) on the table in front of them, and responses were made with the right hand on an external keyboard. Participants completed 10 practice trials, and were required to repeat these until ≥80% accuracy was achieved. The main task consisted of three blocks of 36 trials, with each block lasting approximately 4 min.

Left- and right-hand stimuli were a direct mirror of one another along the vertical axis, and allowed the independent manipulation of spatial compatibility of the hand stimuli, that is, manipulation of the spatial location of the observed finger movement independent from finger identity. On each trial, the task-irrelevant hand performed either imitatively compatible or incompatible actions (imitative compatibility manipulation) on the same or different side of space (spatial compatibility manipulation) to the response required by the participant. Hand stimuli in the standard trials were manipulated in a 2 × 2 (imitatively compatibility × spatial compatibility) design. This produced four main trial types with a further two baseline trial types for left- and right-hand stimuli. In an imitatively compatible trial, a participant prompted to lift his or her right index finger may observe a right hand also lifting its index finger (this trial is also spatially compatible), or he or she may observe a left hand lifting its index finger (this trial is spatially incompatible). In an imitatively incompatible trial, the right-hand index finger response may be performed during the observation of a left hand lifting its middle finger, with this trial being spatially compatible with the required right-hand index finger lifting response; or during the observation of a right hand lifting its middle finger, with this trial being spatially incompatible with the required response.

#### Questionnaire measures

Prior to completing the task, participants completed both the Toronto Alexithymia Scale (TAS-20; Bagby, Taylor, & Parker, 1994) and the Autism Spectrum Quotient (AQ; Baron-Cohen, Wheelwright, Skinner, Martin, & Clubley, 2001). Typically, levels of alexithymia, as indexed by the TAS-20, and levels of autistic traits, as indexed by the AQ, are correlated (Aaron, Benson, & Park, 2015), and autistic traits have been predicted to be related to the inhibition of imitation by Quattrocki and Friston (2014). Thus, in the current study we incorporated both measures to identify the variance in performance accounted for by levels of alexithymia over and above that accounted for by autistic traits.

### Results

#### Alexithymia

The mean sample TAS-20 score observed (46.42) was similar to population figures, but with a wider distribution (*SD* = 14.79; population *M* = 45.57, *SD* = 11.35; Parker, Taylor, & Bagby, 2003). AQ scores showed the same pattern (*M* = 18.35; *SD* = 8.74; population *M* = 16.94, *SD* = 5.59; Ruzich et al., 2015).

#### Imitation-inhibition task

**Figure 2 fig2:**
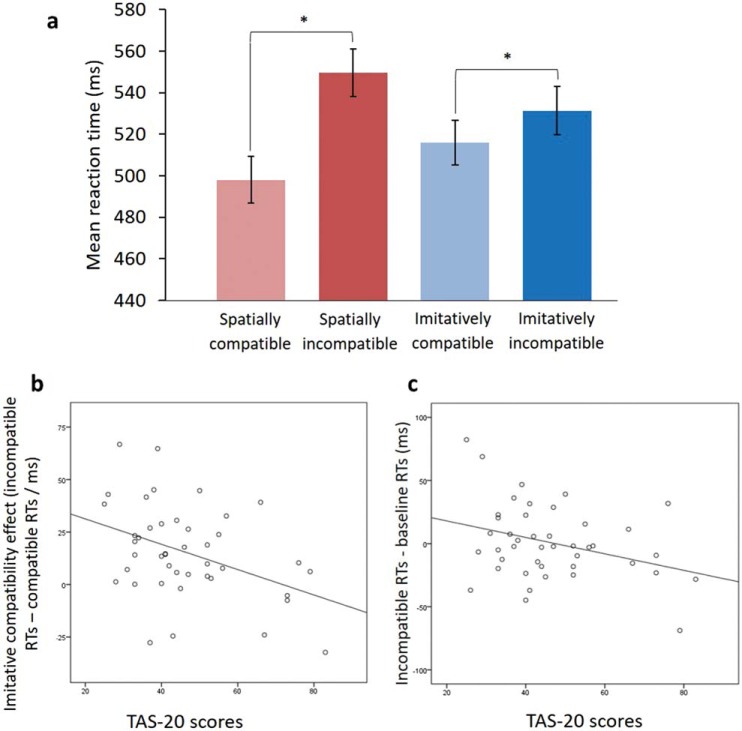
(a) Mean reaction times (RTs; in milliseconds) for each of the four main trial types (spatially compatible, spatially incompatible, imitatively compatible, and imitatively incompatible). Error bars represent the standard error of the mean. Significance at *p* < 0.001 is denoted by *. (b) The relationship between imitative compatibility effects (incompatible RTs—compatible RTs/ms) and Toronto Alexithymia Scale (TAS-20) alexithymia scores. (c) The relationship between the degree of response slowing on imitatively incompatible trials (imitatively incompatible RTs—baseline RTs/ms) and TAS-20 alexithymia scores. See the online article for the color version of this figure.

Mean RT was calculated for each trial type. RTs for spatially and imitatively compatible trials were faster than their incompatible counterparts (Figure 2a), and consistent with RTs observed previously (Sowden & Catmur, 2015; Sowden et al., 2015). These were analyzed using a two-way, repeated-measures analysis of variance (ANOVA), with within-subject factors of spatial compatibility (compatible, incompatible) and imitative compatibility (compatible, incompatible), which revealed significant main effects of both spatial compatibility, *F*(1, 42) = 136.9, *p* < .001, η_p_^2^ = .77, and imitative compatibility, *F*(1, 42) = 20.57, *p* < .001, η_p_^2^ = .33. There was no significant interaction between spatial and imitative compatibility (*p* = .845).[Fig-anchor fig2]

Simple bivariate correlations revealed that alexithymia (as indicated by TAS-20 scores) was significantly associated with imitative compatibility (*r* = −.404, *p* = .007), whereby as alexithymia scores increased, imitative compatibility decreased (Figure 2b). Spatial compatibility was not associated with alexithymia (*r* = −.168, *p* = .281). When imitatively compatible and incompatible trials were assessed separately (after subtracting RT on baseline trials), only the imitatively incompatible trials were significantly associated with alexithymia (imitatively incompatible trials, *r* = −.325, *p* = .031; imitatively compatible trials, *r* = .028, *p* = .857). As alexithymia scores increased, the degree to which observed incompatible actions interfered with participant responses decreased (Figure 2c). These results are very much in line with those found by Ainley and colleagues (Ainley et al., 2014)—who found a correlation coefficient for the association between interoceptive accuracy and imitation-inhibition of .41—and are thus consistent with the hypothesis that higher levels of alexithymia reflect decreased interoceptive accuracy.

Alexithymic and autistic traits were positively correlated (*r* = .404, *p* = .007), consistent with existing literature. Variables such as age, gender, and overall mean RT also influence performance on basic cognitive tasks (Harms et al., 2010). Thus, hierarchical regression analyses were conducted to test whether alexithymia accounts for variance over and above that explained by these other factors.

Two hierarchical regressions were conducted to model the variance in the size of the imitative compatibility effect and RT on imitatively incompatible trials, respectively. The demographic variables (age, gender, mean RT, and AQ scores) were entered into the first step of the regression models. Neither AQ nor the interaction between AQ and TAS scores were significant in their association with either the imitative compatibility effect or performance on imitatively incompatible trials (*p*s > .05) and first-level models for both regression analyses were not significant (*p* = .249 and *p* = .207, respectively). Alexithymia scores were entered into the second step of the model and revealed alexithymia to be a significant predictor both of the imitative compatibility effect, β = −.650, *t*(42) = −2.81, *p* = .008, and of RT on imitatively incompatible trials, β = −.819, *t*(42) = −2.67, *p* = .011. The addition of alexithymia scores significantly improved the fit of both models, increasing the variance accounted for by 18.5% for the imitative compatibility model, *F*(1, 37) = 7.89, *p* = .008, and by 18.2% for the incompatible trials model, *F*(1, 37) = 7.12, *p* = .011.

### Discussion

The present study sought to investigate the relationship between alexithymia and the ability to inhibit imitation, based on an association between imitation-inhibition and interoceptive accuracy (Ainley et al., 2014) and the hypothesis that alexithymia is characterized by interoceptive impairment (Bird & Viding, 2014; Brewer et al., 2015). Moreover, the specificity of the link between alexithymia and imitation-inhibition was investigated through the use of a task in which imitative and spatial compatibility effects could be dissociated.

Results were as predicted by models suggesting that alexithymia is a product of general interoceptive deficits; increasing alexithymia was associated with improved ability to inhibit imitation in the same way, and to the same degree, as interoceptive accuracy (Ainley et al., 2014). The relationship between alexithymia and imitation-inhibition was specific to imitatively incompatible trials, suggesting that performance was driven by the ability to distinguish and control representations of one’s own motor intention from that of the other, rather than a tendency to imitate. If the association was driven by imitation, rather than imitation inhibition, then one would also expect to see an association between alexithymia and RT on imitatively compatible trials.

Furthermore, the relationship between alexithymia and the inhibition of imitation was specific to inhibition of imitative responses. There was no relationship between alexithymia and spatial compatibility, which is particularly striking as responding on spatially incompatible trials necessitates inhibition of an automatic stimulus-response mapping—a task closely matched to that necessary on imitatively incompatible trials. This finding provides further confidence in the attribution of effects to self- and other-related processing, rather than general executive function or motor inhibition ability.

These findings contribute to our understanding of the relationship among alexithymia, interoception, and social ability. While supporting the link between alexithymia and reduced interoceptive accuracy, they suggest that alexithymia may be characterized by increased imitative control according to task or situational demands. Although imitation in social situations is generally considered to promote affiliation—for example, people who engage in imitative behavior are rated as more likable than those who do not (Chartrand & Bargh, 1999)—humans do not always imitate. Motivational and situational factors play an important role in modulating the extent to which people imitate others. For example, individuals are more likely to engage in mimicry with in-group members if they share a common goal and need to cooperate, but not when they are in competition (LaFrance, 1985; although see Marsh, Bird, & Catmur, 2016). Our results suggest that individuals with alexithymia may be better able to precisely modulate their degree of imitation in such situations, leading to a more selective impact on social relationships.

## Conclusion

The present study supports the suggestion that interoceptive atypicalities observed in individuals with alexithymia may not be selective to interpreting one’s own emotions, but may in fact be associated with more general interoceptive difficulties. This conclusion is of clinical interest, as impaired interoception may provide an explanation for the symptom commonalities seen across a large number of neurological, neurodevelopmental, and psychiatric disorders, all characterized by high rates of alexithymia. Given the link between alexithymia and interoceptive awareness (Herbert et al., 2011; Shah et al., 2016), our findings are consistent with those of Ainley and colleagues, supporting the link between interoceptive accuracy and the inhibition of imitation behavior, while providing an extension to this work by demonstrating the specificity of this link to self- and other-related processing rather than general executive function or motor inhibition. These findings are significant for our understanding of the specificity of interoceptive and behavioral profiles of individuals with alexithymia, but may also necessitate revision of current theoretical models of the relationship between interoception and sociocognitive ability.
